# Ten-year experiences with Tracheostomy at a University teaching hospital in Northwestern Tanzania: A retrospective review of 214 cases

**DOI:** 10.1186/1749-7922-6-38

**Published:** 2011-11-10

**Authors:** Japhet M Gilyoma, Darius D Balumuka, Phillipo L Chalya

**Affiliations:** 1 Department of Surgery, Weill-Bugando University Colleges of Health Sciences, Mwanza, Tanzania

**Keywords:** Tracheostomy, Indications, Outcome, Tanzania

## Abstract

**Background:**

Tracheostomy remains a very important life saving surgical procedure worldwide and particularly in our environment where patients present late in upper airway obstruction. Little work has been done on this subject in our environment and therefore it was necessary to conduct this study to describe our own experiences with tracheostomy, outlining the common indications and outcome of tracheostomized patients in our setting and compare our results with those from other centers in the world.

**Methods:**

This was a 10-year retrospective study which was conducted at Bugando Medical Centre from January 2001 to December 2010. Data were retrieved from patients' files kept in the Medical record department and analyzed using SPSS computer software version 15.0. Ethical approval to conduct the study was obtained from relevant authority before the commencement of the study.

**Results:**

A total of 214 patients were studied. The male to female ratio was 3.1: 1. The majority of patients were in the 3^rd ^decade of life. The most common indication for tracheostomy was upper airway obstruction secondary to traumatic causes in 55.1% of patients, followed by upper airway obstruction due to neoplastic causes in 39.3% of cases. The majority of tracheostomies (80.4%) were performed as an emergency. Transverse skin crease incision was employed in all the cases. Post-tracheostomy complication rate was 21.5%. Complication rate was significantly higher in emergency tracheostomy than in electives (*P *< 0.001). The duration of temporary tracheostomy ranged from 8 days to 46 months, with a median duration of 4 months. Tracheostomy decannulation was successively performed in 72.4% of patients who survived. Mortality rate was 13.6%. The mortality was due to their underlying illnesses, none had tracheostomy-related mortality.

**Conclusion:**

Upper airway obstruction secondary to trauma and laryngeal tumors still remains the most common indication for tracheostomy in our centre and tracheostomy is still a life saving procedure in the surgical management of airway despite complications which are seen more commonly in paediatric patients. Most of tracheostomy related complications can be avoided by meticulous attention to the details of the technique and postoperative tracheostomy care by skilled and trained staff.

## Background

Tracheostomy, an ancient surgical procedure originally described in the first century BC [1], is one of the more commonly performed surgical procedures in critically ill patients who require prolonged mechanical ventilation, and is predicted to become more common as demand for intensive care services increases [2,3]. Approximately 10% of mechanically ventilated critically ill patients receive a tracheostomy to facilitate prolonged airway and ventilatory support [4-7]. It is a life-saving procedure when performed with an appropriate indication and surgical technique [8,9]. Other methods of airway intervention include endotracheal intubation, cricothyroidotomy, and Percutaneous Dilatation Tracheostomy [10,11].

The most common indications for tracheostomy are relieve of upper airway obstruction, prolonged mechanical ventilation, airway protection in the comatose and facilitation of tracheo-bronchial toileting [11]. There is a changing trend in literature as regarding the indications and outcome of tracheostomy especially in children for the management of the airway [10-13]. In the past, short term tracheostomy for obstructive airway disease secondary to acute inflammatory infection was the most common indication [14] but in recent time trauma to the upper airway has become the commonest indication [10,11]. These have been attributed to the changes in the epidemiology of infectious diseases due to early diagnosis, adequate use of antibiotics and the improvement in the capabilities of medical technology [10,11,15].

Tracheostomy in the pediatric age group has been reported to be different from that in adults because in pediatric patients this procedure is challenging and technically more demanding and carries higher degree of morbidity and mortality when compared to the adult population [16].

The procedure of tracheostomy is associated with numerous complications which may occur anytime during the operative and postoperative periods [17,18]. These complications are more common in emergency tracheostomy than in elective ones [17]. Complication rates associated with tracheostomy have been reported in literature to range from 6 to 66 percent and the mortality rate related to tracheostomy is reported to be less then 2% [18]. Complications and mortality associated with tracheostomy are mostly avoidable if the procedure is carefully performed and the postoperative management strictly and conscientiously adhered to [19].

Despite the fact that tracheostomy is a common life saving procedure in the care of critically ill patients, there is paucity of information in our local setting regarding the subject. The aim of this retrospective study is to highlight our own experiences with tracheostomy outlining the common indications and outcome of patients with tracheostomy and compare our results with those from other centers in the world.

## Methods

### Study design and setting

A retrospective review of patients who had tracheotomies performed at Bugando Medical Centre during a ten-year period between January 2001 and December 2010 was carried out. Bugando Medical Centre is one of the four tertiary and referral hospitals in the country and has a bed capacity of 1000. It is also a teaching hospital for the Weill-Bugando University College of Health Sciences. The hospital has a 12-bed adult and 10-bed paediatric multi-disciplinary Intensive Care Unit (ICU) which is headed by a consultant anesthesiologist and run by trained ICU nurses.

### Study subjects

The study included all patients who underwent tracheostomy at Bugando Medical Centre during the period under study. Patients who had incomplete or missed basic information were excluded from the study. Data were retrieved from patient registers kept in the Medical record departments, the surgical wards, and operating theatre and entered in a preformed questionnaire before analysis. Included in the questionnaire were; demographic profile (age, sex), primary diagnosis, indication for tracheotomy, surgical technique, duration of the tracheotomy before decannulation, hospital stay and outcome of management such as complications, death and cause of death. The primary diagnosis was classified based on the aetiology which is divided into trauma, infection/inflammation, Neoplasm, congenital and others. The indications for tracheostomy were divided into upper airway obstruction, respiratory insufficiency, bronchial toileting, adjunct to head and neck surgeries. Complications related to tracheostomy were classified as: immediate post-operative period (i.e. within the first 24 hours after surgery), early post-operative period (i.e. within the first week after surgery) and late post-operative period (i.e. beyond one week). Tracheostomies were performed in emergency and electively both under general as well as local anesthesia. The procedure was performed under general anaesthesia in the operating theatre and bedside tracheostomy was performed in the intensive care unit (ICU) under local anaesthesia. Transverse skin crease incision was employed in all the cases.

All the procedures were carried out by surgeons, residents or registrars, while trained ward staff carried out postoperative tracheostomy care. An electric suction machine was provided at bedside for suction as needed.

Tracheostomy decannulation was carried out depending upon the etiology and satisfactory maintenance of the airway. All of them were decannulated in the ward. They were kept under observation for 24 hours after decannulation and were sent home. Instruction was given to do air sealed dressing over the stoma, allowing healing by secondary intension. Patient and attendant were educated that if the patient develops respiratory distress he should be brought to the hospital immediately. First follow up was done after two weeks. When no complication was observed at home, then monthly check up for one year depending upon the condition of the patient.

### Statistical analysis

The statistical analysis was performed using statistical package for social sciences (SPSS) version 15.0 for Windows (SPSS, Chicago IL, USA). The mean ± standard deviation (SD), median and ranges were calculated for continuous variables whereas proportions and frequency tables were used to summarize categorical variables. Continuous variables were categorized. Chi-square (χ2) test were used to test for the significance of association between the independent (predictor) and dependent (outcome) variables in the categorical variables. The level of significance was considered as P < 0.05. Multivariate logistic regression analysis was used to determine predictor variables that predict the outcome.

### Ethical consideration

Ethical approval to conduct the study was sought from the Weill-Bugando University College of Health Sciences/Bugando Medical Centre joint institutional ethic review committee before the commencement of the study.

## Results

### Demographic profile

Two hundred and fourteen patients had tracheostomy within the study period. One hundred and sixty-two (75.7%) patients were males and females were fifty-two (24.3%) with a male to female ratio of 3.1: 1. Their ages ranged from 1 year to 76 years with the median and mean age of 36 and 38.34 ± 12.26 years respectively. The majority of patients were in the 3^rd ^decade of life (36.7%).

### Timing, purpose and indications of tracheostomy

One hundred and seventy-two tracheotomies (80.4%) were performed as an emergency while forty-two (19.6%) as elective procedures. Of the 214 tracheostomized patients, 184 (86.0%) had temporary tracheostomy and the remaining 30(14.0%) had permanent tracheostomy as part of their treatment.

The most common indication for tracheostomy was upper airway obstruction secondary to traumatic causes in 55.1% of patients, followed by upper airway obstruction due to neoplastic causes in 39.3% of cases (Table 1). High incidence of traumatic causes of upper airway obstruction was found between the third and fourth decades of life, while the 7-8^th ^decades of life recorded high incidence of laryngeal and other head and neck malignancies. Laryngeal papillomas causing upper airway obstruction were recorded as the most common indication for tracheostomy in the first decade of life.

**Table 1:** Indications for Tracheostomy

Indications	Pathological causes	Frequency	Percentages
**Upper airway obstruction**		**178**	**83.2**
	*Traumatic*	***98***	***55.1***
	- Severe head injuries	69	70.4
	- Foreign body aspiration	13	13.3
	- Severe maxillofacial injuries	9	9.2
	- Cut throat	7	7.1
	*Neoplastic*	***70***	***39.3***
	- Laryngeal tumors	60	85.7
	- Thyroid cancer	4	5.7
	- Other neck tumors	6	8.6
	*Infections*	***18***	***10.1***
	- Tetanus	16	88.9
	- Retropharyngeal abscess	2	11.1
	*Congenital lesions*	***2***	***1.1***
	- Laryngeal web	1	50.0
	- Laryngeal stenosis	1	50.0
**Mechanical ventilator support/** **Tracheobronchial toileting**		**26**	**9.3**
	Prolonged ventilation	24	92.3
	Diaphragmatic Injury	2	7.7
**Adjunct to head and neck surgeries**		**4**	**1.9**
	Anticipated difficult intubation	4	100.0
			
**Others**		**6**	**1.9**
	Post-thyroidectomy tracheomalacia	3	50.0
	? Gullein Barre syndrome	1	16.7
	Failed endotracheal intubation	1	16.7
	Cause not established	1	16.7

In patients who had tracheostomy secondary to prolonged ventilation, the duration of intubation before tracheostomy was performed ranged from 4 to 62 days with a median duration of 26 days.

The vast majority of patients, 197 (92.1%) underwent tracheostomy under general anaesthesia in the operating theatre and the remaining 17 (7.9%) patients underwent bedside tracheostomy in the intensive care unit (ICU). Transverse skin crease incision was employed in all the cases.

### Post-tracheostomy complications

Complications related to tracheostomy were seen in 46 patients giving a complication rate of 21.5%. Of these, 2 (4.3%) occurred in the immediate post-operative period (i.e. within the first 24 hours after surgery), 10 (21.7%) in the early post-operative period (i.e. within the first week after surgery) and 30 (65.2%) occurred in the late post-operative period (i.e. beyond one week). The period of post-operative complications was not recorded in 4 (8.7%). There were no intraoperative complications (Table 2). Post-tracheostomy complication rate was significantly higher in emergency tracheostomy than in elective one (73.9% versus 26.1%) (*P *< 0.001). Complication rate related to tracheostomy was also significantly higher in children aged 10 years and below than in adult patients (*P *< 0.001).

**Table 2:** Post-tracheostomy Complications (N = 46)

Period	Complications	Frequency	Percentage
**Intraoperative**	No complication	-	-
**Immediate complications**	Bleeding	1	2.2
	Subcutaneous emphysema	1	2.2
**Early complications**	Aspiration pneumonia	6	13.0
	Accidental decannulation	2	4.4
	Tracheal tube obstruction	2	4.4
**Late complications**	Suprastomal granulation tissue	17	37.0
	Stomal infection	11	23.9
	Tracheal stenosis	1	2.2
	Impacted tracheostomy tube	1	2.2

### Outcome of tracheostomy

The duration of temporary tracheostomy depended on the primary pathology and ranged from 8 days to 46 months, with a median duration of 4 months. Tracheostomy decannulation was successively performed in 155 (72.4%) patients who survived. Of these, 102 (65.8%) patients were discharged home after decannulation and the remaining 53 (34.2%) were discharged home with their tracheotomies. In the group of patients who were discharged home with their tracheostomies, tracheostomy decannulation was accomplished successively in all patients during subsequent follow up visits. The median period of post-tracheostomy intensive care unit (ICU) stay was 18 days (range: 6-36 days) and median period of hospital stay was 26 days (range: 7-52 days). Thirty-two (14.0%) patients had permanent tracheostomy needed for either curative or palliative management. Twenty-nine patients died giving an overall mortality rate of 13.6%. The mortality was due to their underlying illnesses, none had tracheostomy-related mortality. Follow up of all patients after decannulation was uneventful.

## Discussion

Since it was originally described in the first century B.C [1], tracheostomy remains a life-saving surgical procedure commonly performed in critically ill patients. In this review, the highest age incidence of the patients who had tracheostomy was in the third decade and males were more affected. Similar demographic profile was reported by other workers [10,11,18,20]. Male preponderance in this age group may be due to their increased susceptibility to trauma which necessitated prolonged intubation and assisted ventilation in some of them.

The indications of tracheostomy are diverse and changing. There has been a change in the' indications for tracheostomy over the past two decades [10-13]. In the past, infective conditions such as epiglottitis and laryngotracheobronchitis were major indications for tracheostomy but the better handling of infections with the use of intubation and conservative management in the intensive care unit has reduced the incidence of these indications [14,15]. The most common indication for tracheostomy in our series was upper airway obstruction secondary to traumatic causes followed by upper airway obstruction due to neoplastic causes, which is at variance with other reports which reported carcinoma of the larynx as the most common indication for tracheostomy followed by prolonged ventilation and foreign body aspirations [10]. These variations between series might be due to different patient populations. The commonest indication recorded in the first decade of life in the present study was upper airway obstruction primarily from laryngeal papillomas, which necessitated emergency tracheostomy as these patients presented in respiratory distress as shown in other studies [16,21]. The high incidence of laryngeal papilloma could be because of mother to child transmission of the Human Papilloma virus (HPV) during delivery. Further research in our region is required to substantiate this. In agreement with other studies [11,22], upper airway obstruction secondary to laryngeal carcinoma and other neck malignancies were the main indications for tracheostomy in the 7th-8th decade of life. In our experience, all cases with laryngeal carcinoma and other neck malignancies present late in severe respiratory distress and so an emergency tracheostomy was always performed even before confirming the diagnosis. Higher incidence of laryngeal carcinoma in our series may also be that there is a possible increase in the incidence of laryngeal cancer in our society. Further study is needed in our setting to confirm this observation. Trauma to the head and neck was the leading indications in the 3rd decade of life in our series and interestingly the majority of these injuries were from road traffic crashes especially involving motorcycles which have become a major means of commuter transportation in Tanzania. This group represents the economically active age and portrays an economic loss both to the family and the nation and the reason for their high incidence of head and neck injuries reflects their high activity levels and participation in high-risk activities. The fact that the economically productive age-group were mostly involved calls for an urgent public policy response.

The surgical technique employed in all our patients was the transverse skin crease incision in the operating room. This is the method preferred by us whether it's an emergency or an elective tracheostomy because of the advantage of a better cosmetic result though, the vertical incision has the advantage of running in the line of the trachea and it is easy to perform and less vascular.

The presence of postoperative complications has an impact on the final outcome of tracheostomatized patients. The rate of postoperative complication in our study was 21.5%, which is higher than what was reported by others [10,20]. However, much higher complication rates have been reported from other centres in Nigeria [11,16,18,19]. In other studies, complication rates of between 6-66% have been quoted [20,23]. The reason for high rate of complications following tracheostomy in our study may be because the majority of tracheostomies in our patients were performed on emergency basis by non-otorhinolaryngologist junior doctors who may have little experiences in performing these procedures. It is therefore recommended that tracheostomy should be performed by an experienced surgeon with adequate facilities to reduce the potential complications. Post-tracheostomy complication rates were found to be significantly higher in emergency tracheostomy than in elective one, which is comparable to other studies done elsewhere [16,19]. This observation is at variance with one report which reported elective tracheostomy as the most frequent performed procedure [20]. Complication rates related to tracheostomy was also significantly higher in children aged 10 years and below than in adult patients which is in agreement with other studies [16,20]. High complication rate in patients who had emergency tracheostomy can be explained by the fact that the majority of patients with upper airway obstruction presented late to the Accident and Emergency department in severe respiratory obstruction and so emergency tracheostomy was always a rule. High rate of complication among children aged 10 years and below is attributed to the fact that tracheostomy in children is challenging and technically more difficult due to small caliber of their larynx and trachea and therefore carries higher postoperative complication rate when compared to the adult population. In the present study, suprastomal granulation tissue and stomal infection were found to be the most common complications of tracheostomy. Similar finding were also reported by Fasunla *et al *[24]. Complication rates associated with tracheostomy can be prevented by good surgical technique and meticulous postoperative care. Suprastomal granulation tissue is a notable late complication of tracheostomy that can be prevented with good surgical technique, sparing the cricoid cartilage during dissection. Stomal infection should be promptly treated and cuffed orotracheal intubation for more than a week in unconscious and tetanus patients should be avoided.

Tracheostomy decannulation in patients with temporary tracheostomy was successfully carried out in 72.4% of patients who survived, which is almost similar to the study done by Hussain *et al *[25] showing 74.1% decannulation accomplished successfully. The optimal timing of tracheostomy decannulation in patients with temporary tracheostomy depends mainly on the underlying disease and should be considered only if the original upper-airway obstruction is resolved, if airway secretions are controlled, and if mechanical ventilation is no longer needed [26].

The overall mortality recorded in our series was 13.6% and these were from underlying diseases. There was no mortality attributed to tracheostomy in this present review reflecting significant improvements not only in the skill of placing a tracheostomy but also in the post operative management of these patients in our hospital.

Our figures for the overall median duration of hospital stay in the present study was 26 days, which is higher compared to what is reported in other studies [10,11]. The reasons for the longer duration of hospital stay may be attributed to the underlying disease and presence of postoperative complications. Also, despite being a life-saving procedure, tracheostomy is not psychosocially acceptable to most patients because of the difficulty with phonation and the stigma associated with it by some uninformed people. Therefore, most patients with temporary tracheostomy desire decannulation before being discharged into the community from the hospital. This might have contributed to longer duration of hospital stay in this study. Due to the poor socio-economic conditions in our setting, the duration of inpatient stay for our patients may be longer than expected due to social reasons.

The potential limitation of this study is that it is retrospective from a single centre and the fact that information about some patients was incomplete in view of the retrospective nature of the study might have introduced some bias in our findings. A similar study in a prospective setting is highly recommended in order to describe our experiences of tracheostomies not only in our centre but also country-wide.

## Conclusion

Upper airway obstruction secondary to trauma and laryngeal tumors still remains the most common indication for tracheostomy in our centre. Tracheostomy is still a life saving procedure in the surgical management of airway despite complications which are seen more commonly in paediatric patients. Most of complications related to tracheostomy can be avoidable by meticulous surgical technique and postoperative tracheostomy care by skilled and trained staff.

## Competing interests

The authors declare that they have no competing interests.

## Authors' contributions

JMG conceived the study and did the literature search, coordinated the write-up, editing. PLC participated in the literature search, writing of the manuscript, editing and submission of the article. All the authors read and approved the final manuscript

## Authors' information

JMG: Senior Consultant General/ENT surgeon, Senior Lecturer and Head, Department of Surgery, Well Bugando University College of Health Sciences. PLC: Consultant general surgeon and Senior Lecturer, Department of Surgery, Well Bugando University College of Health Sciences.
